# Exploring the GMO narrative through labeling: strategies, products, and politics

**DOI:** 10.1080/21645698.2024.2318027

**Published:** 2024-02-25

**Authors:** Camille D. Ryan, Elizabeth Henggeler, Samantha Gilbert, Andrew J. Schaul, John T. Swarthout

**Affiliations:** a Strategic Insights, Bayer Crop Science Canada,Calgary, Canada; b Strategic Insights, Bayer Crop Science, St. Louis, MO, USA; c E-Commerce Search and Catalog Analysis, Millipore Sigma, St. Louis, MO, USA; d Regulatory Scientific Affairs, Bayer Crop Science, Chesterfield, MO, USA

**Keywords:** Disinformation, genetically modified organisms, GMOs, labeling, misinformation, public perceptions, right to know, social media

## Abstract

Labels are influential signals in the marketplace intended to inform and to eliminate buyer confusion. Despite this, food labels continue to be the subject of debate. None more so than non-GMO (genetically modified organisms) labels. This manuscript provides a timeline of the evolution of GMO labels beginning with the early history of the anti-GMO movement to the current National Bioengineered Food Disclosure Standard in the United States. Using media and market intelligence data collected through Buzzsumo™ and Mintel™, public discourse of GMOs is analyzed in relation to sociopolitical events and the number of new food products with anti-GMO labels, respectively. Policy document and publication data is collected with Overton™ to illustrate the policy landscape for the GMO topic and how it has changed over time. Analysis of the collective data illustrates that while social media and policy engagement around the topic of GMOs has diminished over time, the number of new products with a GMO-free designation continues to grow. While discourse peaked at one point, and has since declined, our results suggest that the legacy of an anti-GMO narrative remains firmly embedded in the social psyche, evidenced by the continuing rise of products with GMO-free designation. Campaigns for GMO food labels to satisfy consumers’ right to know were successful and the perceived need for this information now appears to be self-sustaining.

## Introduction

Nearly 90% of stimuli perceived by consumers reaches them by visual means.^1^ This includes food labels which act as “extrinsic cues that can assist consumers inferring product quality and forming quality expectations”^2^ p. 454). In addition to attracting attention through aesthetics, labels are important for product differentiation, brand distinction, and for information about features or ingredients.^2–5^ Collectively, the visual appeal and content of labels influence purchasing decisions and increase market share for labeled products, providing economic gain for food companies.^2,3,6^

In 1973, the Food and Drug Administration (FDA) proposed changes to labeling requirements “in an effort to eliminate buyer confusion” in food purchases^7^ p. 385). These requirements were intended to “reflect a changing perspective of consumer rights and seller responsibilities concerning product information”^8^ p. 14). The labeling of food products has been the subject of constant debate with a range of viewpoints, from advocating for the consumers’ “right to know” to pointing out the costs and/or cognitive limitations of processing extraneous or non-meaningful information.^8^ While the “labeling war” (as characterized by^7^) has raged on over the last few decades, labels – the arguments for and against – have evolved. Today, labels are often tailored to mirror consumers’ opinions on various environmental and sustainability issues, or appeal to other lifestyle factors.^5^ Examples including “gluten-free,” “non-fat,” “low sodium,” “zero cholesterol,” or “GMO-free” may appeal to some customers. This mirroring of lifestyle factors can influence purchasing decisions and increase market share and provide economic gain for food companies.^5^

With respect to GMO-free labels, genetically modified organisms (GMOs) are defined as organisms where genetic material has been altered using genetic engineering (GE) techniques.^9^ Terms like “Biotech,” “GMO” and “genetically engineered” are used by advocates of the technology but critics almost exclusively use “GMO” because of the public’s lack of familiarity with the technology.^10^ Currently, there are 12 GMO crop types on the market in the US.^a^^a.^Alfalfa, apple (Arctic™ varieties), canola, corn, cotton, eggplant, papaya, pineapple (PinkGlow™ Rose), potato, soybean, squash, and sugar beets Other notable GMO crops: Bt brinjal (eggplant) was commercialized in 2013 and is grown in Bangladesh and disease-resistant varieties of cassava are being developed for Africa. A drought tolerant wheat is expected to be released in Brazil later this year. While steps have been made for commercial production of GoldenRice in the Philippines, opposition by activists continue to create roadblocks for its release into the market. AquAdvantage® salmon was approved for human consumption in 2021 but continues to be the subject of controversy. The company recently announced that it will discontinue production at its Canadian plant.

It takes almost $140 million (USD) and up to 13 years to bring a genetically engineered trait to market.^11^ A recent report,^12^ suggests that while the cost of discovery, development, and authorization of a new GM trait has declined by $21 million over the last 10 years, the time for these innovations to reach the market has increased by more than three years. To date, more than 3000 scientific studies have affirmed the safety of these crops in terms of human and environmental impact and almost 300 technical and scientific institutions around the world (including the American Association for the Advancement of Science (AAAS), the European Food Safety Authority (EFSA), the Royal Society of Science, and the World Health Organization) recognize the safety and benefits of GM crops.^13–15^

Despite this consensus on safety and that GMOs undergo stringent regulatory approvals and safety testing all over the world, there continues to be social and political controversy and misinformation about the safety of food derived from genetically engineered crops.^16–23^ GMO is employed as a “dubious meme” used as a target for determined opposition by many activist groups^23^ and used in efforts to purposely sow dissenting positions concerning [GM] crops in the United States.^16^

Furthermore, there is a notable gap or disconnect between public perceptions and the science and safety of GMOs.^24^ In a survey published by the Pew Research Institute in 2015, results demonstrate that while 88% of scientists surveyed believe that genetically modified foods are safe to eat, only 37% of the general public stated that they are safe to eat.^25^ A more recent survey reveals public perception that GMOs are unsafe continues to increase^26^ even though the engagement around the online GMO narrative in media has diminished over time.^21^

In the current study, we seek to build upon the data and analysis of the GMO narrative from earlier work,^21^ to investigate the historical trajectory of GMO labeling, and gain insights into the connection between and among: (1) The factors, actors and events that drive narratives about GMOs; (2) how consumer perceptions of GMOs are portrayed by different groups, (3) the progression of the GMO narrative in policy documents and; (4) the trajectory of GMO labeled products. It is anticipated that the narrative around GMOs as measured by both the volume of articles online and the number of documents in the policy space will dimmish over time, while the non-GMO product market will continue to grow.

To pursue this, we combine qualitative research (background, history, events, actors, etc.) with data analysis to elucidate the history of GMOs, the trajectory of anti-GMO activism, follow-on labeling regimens and explore the history of GMO labeling which culminates in the current National Bioengineered Food Disclosure Standard (NBFDS) (hereafter referred to as “US NBFDS”). Specifically, this study:

1. Illustrates the course of online dialogue and engagement activity around the topic of GMOs including key legislative/political factors and events;
2. Provides a chronological timeline of the GMO labeling narrative within the broader narrative of GMOs from “right to know” to industry-driven labeling;
3. Presents data on the topic of GMOs in policy documents delineated by source and time, and;
4. Presents data illustrating the trajectory of GMO labeled products introduced to the market over time.

## Methods

### GMO Narrative in Social Media

BuzzSumo™ was used to capture data pertaining to the GMO narrative using defined search terms for the GMO topic.^b^^b.^BuzzSumo™ (BuzzSumo, Brighton and Hove, UK). Data Collection Search String: “gmo OR gmos OR ‘genetically modified’ OR ‘genetically modify’ OR ‘genetic modification’ OR ‘genetically engineer’ OR ‘genetically engineered’ OR ‘genetic engineering’ -bitcoin -internet -bacteria -animal -embryo -HIV” Terms preceded by a “-” were specifically excluded from the data collection to reduce irrelevant data. The data set encompasses 117,654 unique online articles from 19,610 unique websites collected between March 2009 and September 2020. This dataset, including articles and associated social media engagement metrics, was used to describe the GMO topic and social media engagement in online media (news, blogs, and websites). As mentioned, article data was collected from 19,610 unique websites and was not a list of sites defined by the authors. Rather these include all sites available via the BuzzSumo™ platform at the time of collection. For example, data was collected from sites ranging from mainstream news (e.g., Yahoo.com and Reuters.com) to advocacy websites (e.g., GeneticLiteracyProject.org and NaturalsNews.com).

An analysis of these data examines the total number of articles and social media engagement with these articles over time. The social media engagement metric from BuzzSumo™ leveraged for analysis was Total Engagements for each article result. Total Engagement refers to the sum of the number of shares, likes, and comments the article URL receives on Facebook, the number of tweets, and retweets containing the article URL on Twitter, the number of shares of the article URL on Pinterest, and the number of Reddit engagements, which are a sum of upvotes and comments, subtracting any downvotes on posts including the article URL.

### GMO Narrative in Policy Documents

Data was collected from Overton™ to illustrate the policy landscape for the GMO topic over time. Overton™ is on online, searchable collection of over 8 million policy documents, parliamentary transcripts, government guidance and think tank research collected globally from 180+ countries. Overton™ defines policy documents very broadly as documents written primarily for or by policymakers that are published by a policy focused source. Policy source types include governmental agencies (e.g., Environmental Protection Agency (EPA) and European Food Safety Authority (EFSA)), intergovernmental organizations ((IGOs); e.g., United Nations (UN) and World Health Organization (WHO)), and think tanks (e.g., Pew Research Center and Consultative Group on International Agricultural Research (CGIAR)).

The data set encompasses 45,994 policy documents collected between March 2009 and 2023 sourced from policy research platform Overton™ using defined search terms for the GMO (see footnote^2^). An analysis of these data examines the total number of policy documents referencing the GMO topic between the dates selected. Search results were categorized in Overton™ by year published, country of origin, document type and source type. Data was exported and is represented graphically to show change in number of documents published by year, country of origin and policy source type.

### Trajectory of GMO-Free Labelled Food Products

Mintel™ was used to collect data to illustrate the trajectory of GMO-Free labeled products introduced to the market over time. The analysis includes data from 95,367 unique food products published between January 1996 (the start of available data) and August 2020 that contained GMO-Free claim on the label. Food label data was collected by Mintel™ in the Global New Products Database (GNPD), a database of newly released consumer packaged goods in 86 markets for 46 categories. The dataset was created by selecting the “GMO-Free” label claim for the Super-Category that matches “Food.” The “GMO-Free” label selected is determined by Mintel based on the food product packaging, which includes product descriptions, claims and labels on the food product packages. The search terms for all food products were “where Super-Category matches Food and Date Published is between 1996-01-01 and 2020-06-30. The search terms for GMO-Free products were “where Super-Category matches Food, and Claims matches GMO-Free as the claim, and Date Published is between 1996-01-01 and 2020-06-30.”

Searches conducted in BuzzSumo, Mintel and Overton were performed in English only and no regional and country geographic filters were used. English only searching may result in regional underrepresentation of policy documents. For example, EU state level policy documents. BuzzSumo captures all data pertaining to the GMO narrative using defined search terms for the GMO topic (see footnote 2). Regarding geography, Overton locates all United Nations documents in the US as the United Nations are headquartered in NYC. This may lead to an overrepresentation of US based articles. Mintel data is US only.

## Results

### BuzzSumo Data: Total Unique Articles and Aggregate Shares

**Figure 1b.**
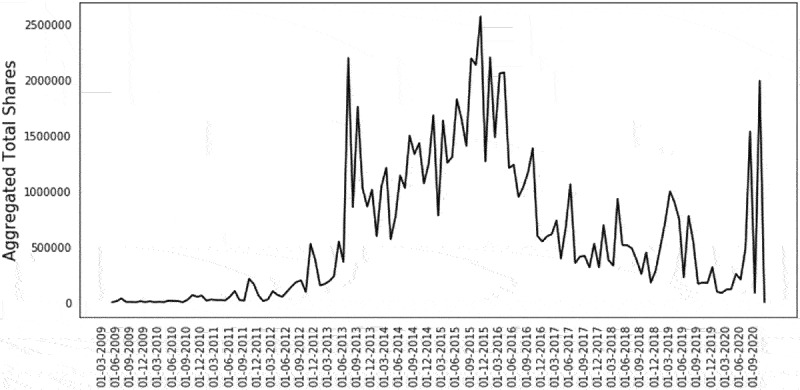
Aggregated total engagements per month.

The full dataset collected for this analysis is presented as the total unique article URLs (Figure 1a) and total engagement of each article URL in the full BuzzSumo™ dataset aggregated at monthly intervals (Figure 1b). The data shows a general trend upward in the online dialogue and engagement beginning in June 2011. While causality cannot be established, the timing of this increase correlates with the Prop 37, California’s ballot initiative for the Mandatory Labeling of Genetically Engineered Food which was voted on in 2012.^27^ Various peaks are evident with the aggregated Total Engagements peaking around September 2015 and then steadily declining to a low point in May of 2020. The decline in both the online article publishing and engagement activity pertaining to the topic of GMOs continues into 2020. Factors contributing to this decline could include a perceived resolution of GMO labeling in the US with US NBFDS, the COVID-19 pandemic, political events globally, a shifting focus on crop protection over biotechnology in food, among others.

Looking at total number of articles, two peaks, one in 2017 and another in 2019 are observed (Figure 1a). Further analysis revealed that the peak in 2017 is comprised of press releases of GMO industry market reports (~400 articles) and the second peak in 2019 is driven by articles about GM salmon and the deactivation of Import Alert 99–40 by the FDA enabling the sale of food derived from GE salmon in the U.S.^28^**Figure 1a.**
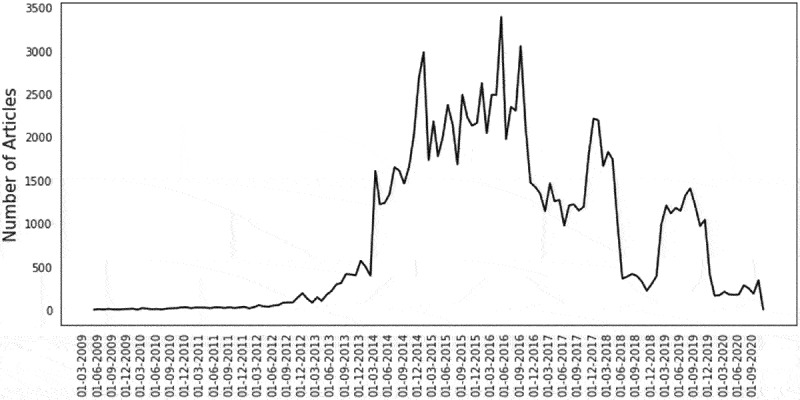
Volume of online articles results per month.

The volume of unique URLs (number of articles) per month as collected from BuzzSumo™ based on search terms (see footnote^2^) are depicted. Date format is Day-Month-Year.

The aggregated total engagements for unique URLs (number of articles) per month as collected from BuzzSumo™ based on search terms is depicted.

Similarly, significant spikes in total engagements are observed after the peak in 2015 (Figure 1b). The prevailing topic of the total engagements from March 2019 – May 2019 pertains to GM salmon articles (31% of volume) and shares from June – Sept 2020 were from GMO mosquito articles (95% of the volume). These topics are noted here but were not removed from the dataset as they are relevant to the topic; directly as GM salmon and to food production in the form of GMO insect driven pest control.

Ryan et al.^21^ identify a potential connection between the (dis)information campaign on GMOs and the establishment of a new marketing approaches including the non-GMO label. An analysis of key legislative measures (e.g., Proposition 37 and Initiative I-522)) with online articles as collected media output on GMOs, illustrated the relationships between events and engagement with online articles. This paper presents a deeper analysis by assessing if key events potentially impact the GMO narrative both in policy documents and social media (Figure 2 a-c). The controversy around the safety of food derived from GMO crops has a long and intricate history involving numerous stakeholders, events, and varied socio-economic outcomes. To gain insights into the connection between consumer perceptions of GMOs and the trajectory of GMO labeled products, a qualitative examination of the history of GMO labeling from the early days of the anti-GMO movement to the evolution of US NBFDS (the National Bioengineered Food Disclosure Standard (NBFDS)) was performed. The analysis details the major events between 1990–2010 (timelines 1990–1999 and 1999–2010; Figure 2a and 2b) which are considered to have shaped and been part of the anti-GMO narrative.^16,21,23,29–31^ A third timeline (Figure 2c) covers the major events (2011–2022) culmination in the fully compliant NBFDS.

### Timeline: Shaping the Anti-GMO Narrative (1990–1999) (Figure 2a)

A signature event in the 1990s was the Calgene “Favr Savr” tomato (see Figure 2a). The tomato was first genetically engineered food approved for release in 1994 and is viewed by some as the driver for the rise of the counter GMO narrative in Europe and other parts of the world.^19^ Other historical events have since intensified the controversy around GMOs (Figure 2a). The bovine spongiform encephalopathy (BSE) crisis of the late 1990s, for example, irreparably damaged the tenuous relationship between the public and food safety organizations in the UK and beyond.^32,33^ In the late 1990s, Friends of the Earth organized a campaign against the introduction of GM foods in supermarkets in the EU.^17^ In the absence of regulations around GM food at the time, retailers were pressured to act, purportedly on behalf of the consumer. In March 1999, Marks & Spencer become first UK supermarket to ban GE ingredients. This set off domino effect for Sainsbury, Safeway, Tesco^34^ many of which took collective action by establishing campaigns (e.g. “GM Free Working Group,” “The Consortium to eliminate GE ingredients from own-label foods,” etc.).^35^**Figure 2a.**
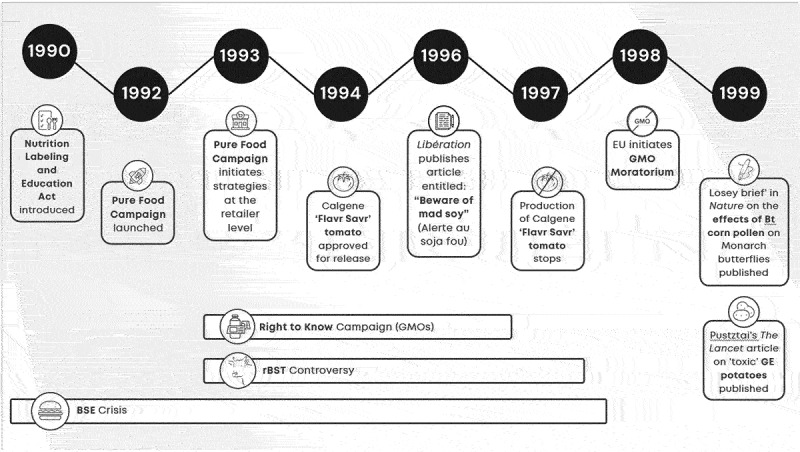
Timeline 1990–1999: shaping the GMO narrative.

**Figure 2c.**
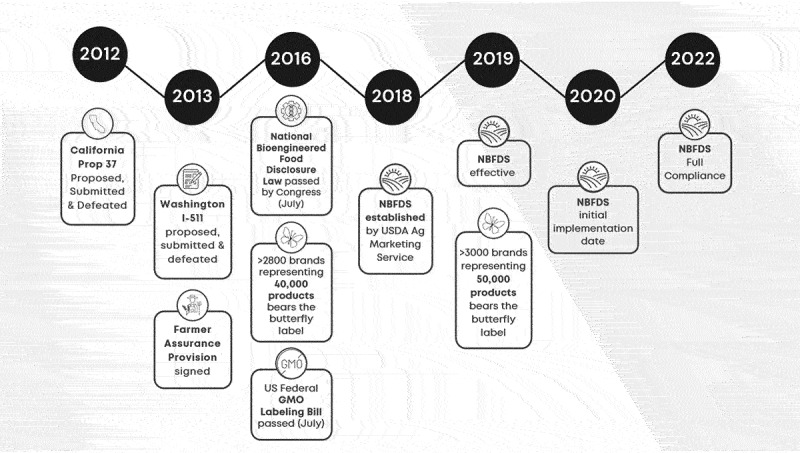
Timeline 2012–2022: creating a new standard.

In the US, and citing business and product-related factors, Calgene stopped production of its genetically engineered Flavr Savr tomato in 1997. In late 1999, McCain Foods, the world’s largest frozen potato processor and a buyer of PEI potatoes, announced that it would not purchase GM potatoes for processing due to perceived consumer resistance.^20^

The right to know campaign in the context of GMOs was adopted in the early 1990s and was driven largely through messaging and actions of the non-governmental organizations (NGOs) such as Greenpeace International, Friends of the Earth International, and the Center for Food Safety.^17^ According to Paarlberg,^17^ the campaigns against GMO technology and foods succeeded because they succeeded first in the EU. A series of events and communications (mainstream articles) originating in the UK and the broader EU collectively helped to shape and influence the global anti-GMO movement for the coming decades^18,21,36,37^ (Figure 2a). In 1998, the EU initiated its 6-year moratorium on GMOs, with two biotech carnation varieties being the last live GMOs plants to win EU approval.^38^ In scholarly spaces, Pustztai’s *Lancet* article on “toxic” GE potatoes in 1999^39^ and then Losey published his “brief” on the effects of Bt corn pollen on Monarch butterflies in 1999.^40^ In the late 1990s and into 2000, the Bovine spongiform encephalopathy (BSE) issue, which started in 1986, received renewed attention as a public health crisis with incidences of deaths reported from its human equivalent, Variant Creutzfeldt-Jakob disease (vCJD).^41^ Collectively, these events cultivated a specious connection between the BSE crisis and GMOs which had been firmly planted in the public discourse. This perpetuated a growing distrust of the institutions linked to these issues.^42^ Another important component of the history of GMO labeling and associated narratives includes Recombinant Bovine Somatotropin (rBST). Recombinant Bovine Somatotropin (rBST), a nearly identical replica of a naturally-occurring growth hormone (BST or BGH) used in dairy cattle,^43–45^ was introduced to the market by Monsanto Company Inc (St. Louis, Missouri) upon its approval by the FDA in 1993. The product was met with skepticism. Headlines like “Crying over Unnatural Milk”^46^ or “Udder Insanity!”^47^ captured attention and the imagination of the public.

### Timeline: From ‘Right to know’ to Rent Seeking (1999–2010) (Figure 2b)

Labeling for “right to know” has been the battle cry even in the early days of rBST. Mark Kastel, a spokesperson for the Wisconsin Farmers Union, encouraged members to label their milk and stated in an interview in 1993, “We’re willing to accept that this product is proven safe. We’re not arguing with that … What we are arguing for is the consumers’ right to know … .”^48^ Of course, the collective FDA and scientific position early in the issue was that there was no material difference in nutrition, composition or safety between GMO foods and their convention counterparts. Thus, there would be no reason to label these foods in a way that would unnecessarily distinguish them from their conventional counterparts.

A significant event in the early 2000s was the adoption of The Cartagena Protocol to the UN Convention on Biological Diversity, an international agreement to ensure the safe handling, transport and use of living modified organisms (LMOs) resulting from modern biotechnology, was adopted in January of 2000. It entered into force in September of 2003 (see timeline 2, Figure 2b). Additionally, in February 2000, the European Commission had adopted communication on the precautionary principle (The Principle) as part of a structured approach to the analysis of risk and risk management.^49,50^

**Figure 2b.**
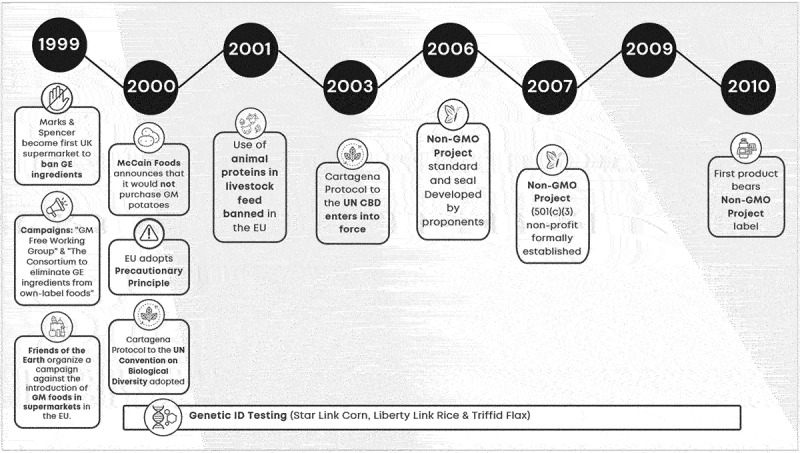
Timeline 1999–2010: from ‘right to know’ to rent seeking.

Responding to growing consumer pressures and the call for mandatory labeling requirements in Europe and Asia, US farmers began to set up “identity preserved markets,” as a way to segregate GM from non-GM commodities.^51^ The Protocol did not address the issue of domestic food labeling explicitly, but it did establish standards for trade on LMOs. Identification of shipments for traceability drove the establishment of domestic labeling regimes. Again, the European Commission and other like-minded countries insisted on labeling to facilitate transparency and traceability.^52^

The rise of international agreements around GMOs (e.g. Cartagena Protocol on Biosafety (CPB)) and the consumer push for “right to know,” provided the foundation for new markets for a number of actors, in particular, the upsurge of GMO testing. Genetic ID (GID) (now FoodChain ID) was established in 1996 and pioneered GMO testing and discovery. The objective for right to know is entangled with the GMO testing and discovery industry. Historically speaking, Genetic ID tested for the genetically engineered corn (StarLink) that led to the recall of over 300 consumer products in 2000. Additionally, Genetic ID discovered the unapproved LibertyLink rice in 2006, as well as Triffid flax which was found in stores of flax in the EU in 2009.^20,22^

Through testing to find genetically engineered ingredients and then by (negative) labeling for market purposes, “Non-GMO Project activists capitalized on this vulnerability by offering retailers a new way to attract consumers and publicly reaffirm their corporate philosophies”^53^ p. 357). In late 2006, purpose was reframed from living up to consumer expectations and protecting human and environmental health, to providing a competitive edge to companies in an increasingly crowded food market.^54^ So, while the argument for labeling appears to largely focus on “right to know,” an often underlying and overlooked objective is an economic one. Clark, Ryan, and Kerr^55^ p. 184) explore Prop 37, California’s ballot initiative for the Mandatory Labeling of Genetically Engineered Food, and its signal for renewed interest in GMOs in 2012. The authors state that “a large, vested interest has arisen in the form of a contingent of the organic industry” (p. 184). Prop 37 was only one of similar ballot initiatives in the US. Through these ballots, consumer awareness is raised, and more consumers may choose to purchase organic (or non-GMO or GM-free) products: “[L]abeling may increase awareness to the benefit of the organic industry … ”^55^ p. 184). Similarly (and years before) Runge et al.^45^ recognized that voluntary labels provide a foundation for the creation of new niche markets (non-GMO and GMO products and seeds).

As Roff^53^ p. 351) outlines, certification projects like the non-GMO verification project, become “vulnerable to industry capture” as marketing tools. Roff further elucidates how the relationship between testing/measurement (Genetic ID) and management/marketing (Non-GMO Project) advanced the “right to know” grassroots endeavor into one driven by rent-seeking: “For Genetic ID, the Non-GMO Project was an opportunity to expand its clientele beyond companies exporting to the European Union and Asia and stimulate a domestic market for non-GMO products for which it would be the principal certifying body. Indeed, although testing is decentralized, Food Chain Global Advisors (FCGA), GID’s parent firm, maintained control of verification and certification. With GID’s guidance, the ‘People Want to Know’ campaign was re-christened The Non-GMO Project and the group released an initial standard and shopping cart seal in late summer of 2006”^53^ p. 357).

The most ardent and influential critics of genetic engineering and GMOs view labeling as a means to an end; with objectives to either elevate the organic industry and products to outright bans on GMOs or both.^56^ For example, the Campaign Director for GMO Inside/Green America is reported as saying: “Our objective is to eliminate GMOs [from the US food supply] but we also see [mandatory] GMO labeling as a useful tool in the meantime because we know that transitioning to a non-GMO supply chain will take time”(cited in^57^).

### Timeline: Creating a New Standard (2012–2022) (Figure 2c)

A patchwork of labeling regimes – the result of consumer pressure and legislative initiatives – threatened to further mislead consumers, increase cost of goods, and create unnecessary regulatory barriers for businesses of all types (Figure 2c). The National Bioengineered Food Disclosure Standard (NBFDS) was designed to mitigate that. In December of 2018, US NBFDS was established by the Agricultural Marketing Service. US NBFDS requires food manufacturers, importers and certain retailers and other entities (nationwide) to label or otherwise disclose whether foods offered for retail sale are bioengineered (BE). It is intended to provide a mandatory uniform national standard for disclosure of information to consumers about the BE status of foods. US NBFDS became effective in February of 2019, with an initial implementation date of January 1, 2020, with full compliance as of January 1, 2022.

### Overton Dataset: Policy Publication on GMOs

**Figure 3.**
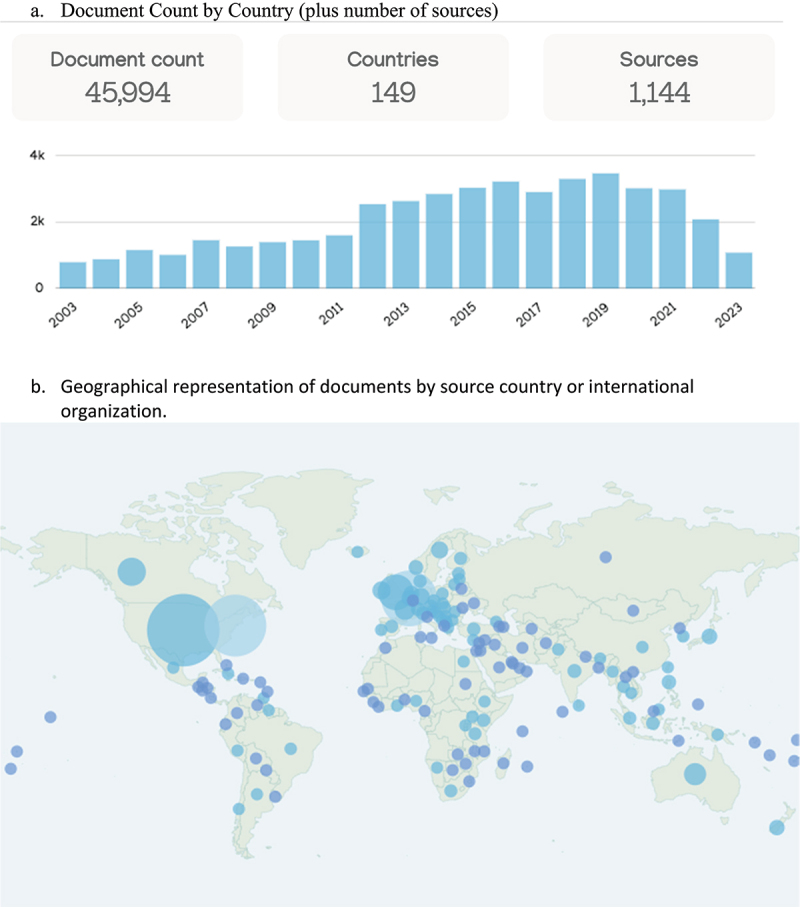
Number of policy documents containing GMO terms. Source: Overton (2011–2023).

The total number of policy documents referencing the GMO topic between 2003–2023 was assessed. Search results were categorized in Overton by year published and country of origin (Figure 3). A total of 45,994 documents from 149 countries and 1,144 different sources were identified. The data shows a general trend upward in total number of policy publications beginning after 2011. Similar to the data for social media engagement, the timing of this increase appears to correlate with the Prop 37, California’s ballot initiative for the Mandatory Labeling of Genetically Engineered Food which was voted on in 2012.^27^ The volume of publication is sustained, peaking between 2014–2021, and declines thereafter. The largest volume of documents originated within the United States, IGOs, and the EU, accounting for 56% (25,581) of all documents.

Search Terms: gmo OR gmos OR “genetically modified” OR “genetically modify” OR “genetic modification” OR “genetically engineer” OR “genetically engineered” OR “genetic engineering” Link to the report page, you will be able to see the Overton screen and diagrams but won’t be able to click into anything.

The scale of the dot represents the number of documents originating in that country or from an international organization, so a country could have two separate circles. For example, there are two large circles in the US map as some large international organizations like the United Nations have headquarters in the US. Color assignments of dots are random and used to help differentiate dots on the map for ease of viewing.

### Mintel Data: Food Products Including a Non-GMO or GMO Free designation

Mintel data was analyzed to assess changes in the growth of products labeled as Non-GMO or with the Non-GMO Project butterfly logo after its introduction in 2010. The total number of unique food products with the GMO-Free label was tabulated from the Mintel dataset (Table 1). The data represents the total number of new products introduced but does not track products removed. Therefore, the total samples of 95,403 represents the accumulated new additions in the dataset but does not reflect the total number of products currently including a Non-GMO or GMO-free designation as part of their label. The data illustrate that snack foods, dairy and bakery items make up the bulk of the products with a non-GMO label representing 48% of the total number of new products added.

**Table 1.** Total number of unique food products with the GMO-Free label from January 1996 through August 2020 (source: Mintel global new products dataset).

Food Category	Total # Products
Snacks	21,484
Dairy	13,100
Bakery	10,806
Sauces & Seasonings	9,637
Processed Fish, Meat, and Egg Products	7,714
Side Dishes	6,409
Breakfast Cereals	4,563
Fruit & Vegetables	3,697
Baby Food	3,633
Chocolate Confectionery	2,891
Meals & Meal Centers	2,581
Desserts & Ice Cream	2,369
Sweet Spreads	1,885
Sugar & Gum Confectionery	1,545
Soup	1,062
Savory Spreads	1,056
Sweeteners & Sugar	971
**Total Sample**	95,403

**Figure 4.**
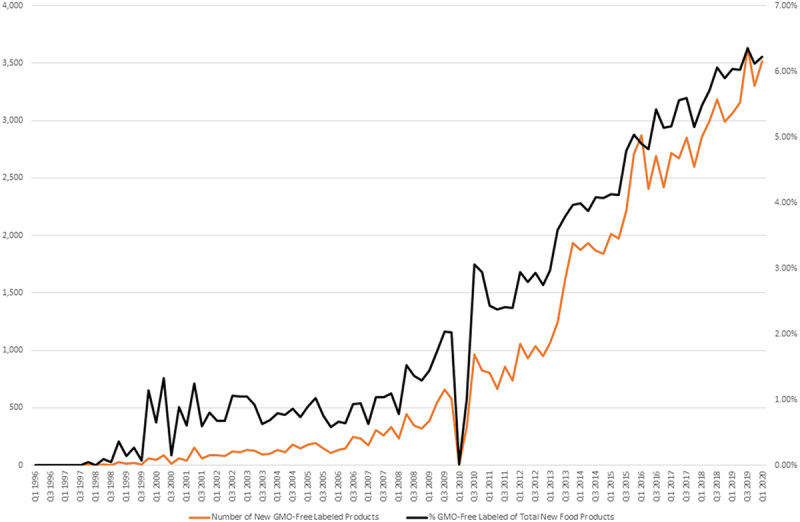
Total volume of labeled food products.

The number of new products with a GMO-free designation continues to increase both in terms of absolute products and percentage or products (Figure 4). As of the first quarter of 2020, a total of 3,514 food products, with a GMO-free label, were introduced. This represents 6.23% of the total of all food products counted by quarter in the Mintel GNDP database. The yearly percentage of non-GMO labeled food from all newly released food went from .8% in 2005, to 1.8% in 2009, and to 6.23% in 2020.

Volume of New Food Products released quarterly with “GMO-Free” label depicting the volume of new food products released per quarter as collected by Mintel.

The decrease in the first quarter of 2010 was investigated further. The dip does not reflect a data collection issue as there was no decrease in the food data that was reviewed. However, the rapid decline coincides with the release of the Non-GMO Project “Butterfly” label in early 2010^58^ and this may indicate that manufacturers delayed changes to labels waiting for the new non-GMO label.

## Discussion

The objective of this study was to gain insights into the connection between consumer responses to GMOs (as portrayed by various parties) and the trajectory of GMO labeled products. To achieve this, we combine qualitative research (background, history, events, actors, etc.) with data analysis to elucidate the history of GMOs, the trajectory of anti-GMO activism, and follow-on labeling regimens. An examination of the history of GMO labeling, from the early days of the anti-GMO movement (Timelines 1 and 2; Figure 2a and 2b) to the evolution of US NBFDS was performed (Timeline 3, Figure 3). Our analysis shows that the history of the anti-GMO movement, leading up to food label lobbying and initiatives, utilized social media to drive negative consumer perceptions about GMOs and this may have created or perpetuated a demand for free-from products. Furthermore, these negative consumer perceptions, demand for consumers’ right to know, and efforts around capturing market share, incited food companies to reimagine how they market products, including adding claims to labels like GMO-free.

The trajectory of the discussion pertaining to the application of GMOs in agriculture and GMO foods peaked in 2016 and has declined through 2020. This decline is reflected both in terms of total number of articles (Figure 1a) and in the aggregated total that published articles are shared over time (Figure 1B). The diminished social media engagement around the topic of GMOs suggests that factions which have problematized GMOs in the past have moved onto other issues. Additionally, much of the remaining social media engagement pertaining to GMOs is no longer focused on GM crops or foods derived from these crops. Rather, the data suggests that the conversation has shifted to other topics like the introduction of GM salmon and release of GM mosquitoes. Unlike the conversation around GM crops and foods derived from these crops, a qualitative review of the sentiment of the media stories pertaining to salmon and mosquitos (data not shown) suggest that public and media perception is largely neutral. Around 17% of the articles, however, including the top shared article on GM salmon (163608 shares), contain a nonsensical word, “Frankenfish,” in the title.

Yet, even as the online dialogue about the application of GMOs in agriculture and GMO foods has diminished, products bearing a non-GMO or GMO-free designation continue to be introduced to the market (Figure 5). This is true in terms of the total number of products bearing the label and the percentage of products bearing a GMO-free designation respective to all products.

One potential reason for the continued growth of products bearing a GMO-free designation is the consumer belief that GMOs are less healthy than non-GMOs or that GMOs are unsafe. PEW research data finds that 51% of U.S. adults believe GMOs are worse for people’s health than non-GMO foods and that this perception continues to be on the rise. Alternatively, PEW also reports that while half of U.S. adults are wary of health effects of genetically modified foods, many also see advantages.^59^ Regarding the question of safety, a PEW survey of people from 20 countries around the world showed that while 48% of people think GMOs are unsafe only 13% felt they were safe to eat.^60^ In the US, 38% of consumers feel GMOs are unsafe compared to 27% who feel they are safe. Interestingly, 33% of US consumers do not know enough about GMOs to say either way. This doubt about or uncertainty around GMO safety is despite the scientific evidence supporting GMO safety.^61^

This distrust in GMOs stems from grassroot efforts purporting that GMOs are unsafe. Grassroot efforts led by a network of individuals and organizations are very powerful structures that can capitalize on scale and leverage the human collective.^62^ This is even more pronounced in our age of mass communication and social media.^21^ Grassroot campaigns around GMO safety preceded the rise in the number of products labeled “non-GMO” or with the non-GMO verified “butterfly” after its introduction in 2010 (See Figure 2b). In the context of GMO labels, the grassroots initiative for labeling for right to know was disrupted and reframed to “People Want to Know” by the Non-GMO Project in 2006.^53^ The right to know endeavor went from living up to consumer expectations for protecting human and environmental health, to a corporatized model that provided a competitive edge to food companies who wished to differentiate in an increasingly crowded food market.

The No to labeling side (including food companies and seed companies that were part of the Grocery Manufacturers Association (GMA)) organized in response to Prop 37, mobilizing $46 million to be spent on messaging around an economic argument to influence voters in advance of the poll.^55^ Prop 37 was ultimately defeated (Figure 3). Yet, beyond 2012 and Prop 37, this “powerful food lobby” (GMA) eventually lost members^63^ when questions were raised about the industry’s ability to speak with one voice, particularly in light of constantly changing and contradictory demands from consumers. The Campbell Soup Company was the first to defect, followed by Unilever, then Mars, Tyson Foods, Nestlé, Dean Foods, Hershey’s, and the grain giant Cargill, citing philosophical differences and shifts in goals and objectives.^63^ In July 2018, Mars, Nestle, Unilever and Danone went on to form a new alliance: Sustainable Food Policy Alliance. Charlie Arnot, CEO of the Center for Food Integrity, stated that the breakdown, in part, reflects fragmentation in the food industry, starting with the fundamental changes taking place with consumers: “We’re no longer as monolithic as we once were … the emerging lifestyle brands are growing markedly faster than the legacy brands … .”^63^ Caswell^64^ p. 24) recognized this shift in the retailer’s agenda: “Food companies will need to view labeling as an opportunity, not a threat, and devise marketing strategies that work with labeling policies.”

Given this market, the public awareness as measured here in the form of media coverage and engagement along with major social and political events played a role in the creation or growth of this market over time. Furthermore, after significant policy conclusions were made in the United States^21^ this market has become self-sustaining and/or has decoupled from the need for continual media conversation to maintain it. More specifically, the number of new products bearing these labels continue to grow in number and has achieved a steady increasing market share in the absence of a continuing public media conversation (Figure 5-1a).

Prior to the establishment of US NBFDS, a state-by-state collage of labeling regimes arose, with 37 food labeling bills introduced in 21 states as of 2013. However, only Maine, Connecticut and Vermont enacted laws requiring the labeling of foods made with genetically engineered (GE) ingredients. US NBFDS was designed to mitigate the uncertainty created by the patchwork of labeling regimes which threatened to further mislead consumers, increase cost of goods, and create unnecessary regulatory barriers for businesses of all types. USDA Secretary of Agriculture, Sonny Purdue announced on December 20, 2018:

The National Bioengineered Food Disclosure Standard increases the transparency of our nation’s food system, establishing guidelines for regulated entities on when and how to disclose bioengineered ingredients … This ensures clear information and labeling consistency for consumers about the ingredients in their food. US NBFDS also avoids a patchwork state-by-state system that could be confusing to consumers.^65^

While US NBFDS has been met with some criticism,^66–70^ others suggest that mandatory labels can improve attitudes toward GMO foods: “ … simple disclosure, one of the suggestions for US NBFDSs being developed at the federal level, is not likely to signal to consumers that GE foods are more risky, unsafe, or otherwise harmful than before label exposure and might, in fact, do the opposite”^71^ p. 3).

The anti-GMO movement led to the right to know movement which then led to states implementing labeling laws, which then led to the development of the Federal Standard to mitigate a patchwork of legislative measure. Brady^72^ p. 774) states, however, that US NBFDS will face litigation challenges because mandatory disclosure “necessarily implicates First Amendment free speech issues.” Brady further expands upon the paradoxes and complexities: “Litigants challenging compelled disclosures have been urging more stringent standards … [As] a result, courts have been applying higher scrutiny levels, which require more substantial government interests to justify infringements on free speech, especially in the context of public health.”

Currently there are more than 3000 verified brands, representing over 50,000 products that are Non-GMO Project verified and net more than $26 billion in annual sales in North America.^58^ A total of 95,403 products contains some iteration of a non-GMO label (Table 1). A typical traditional grocery stores carries 15,000 to 60,000 SKUs (stock-keeping units) (depending on the size of the store). It is unclear what percentage of this total number of SKUs carry the non-GMO label or the percentage of products that will contain the bioengineered label until after it is fully enforced, and it is also unclear what other challenges US NBFDS may face moving forward.

## Conclusions

Oppositional grassroots activism questioning the safety of GMOs formed the foundation for public perceptions of genetically engineered crops and food products derived from those crops. Previous analysis demonstrated that a small group of alternative health and pro-conspiracy sites utilized social media platforms to deliver mis- and disinformation about GMOs to both capture public interest and to create uncertainly around GMO safety.^21^ Building on that work, this current study shows the conversation on social media surrounding GMOs has waned while the growth of additional product and marketing approaches such as non-GMO verification have emerged.

While a direct causal relationship cannot be made, the hypothesis that consumer fears around GMOs perpetuated by grassroot efforts and political events can create new market opportunities, in the form of GMO-free labeled products, appears to be supported. Thus, consumer fears and uncertainty around GMOs, largely created through organized mis- and disinformation campaigns,^16,21^ can be shaped into market opportunities such as free-from claims on food labels.

It is important to note that some labels have a legitimate and informative place in the market. For example, nutrition and ingredients labels or labels that identify food allergens provide important product information for consumers. However, labels containing valid health information are vastly different than labels that declare unproven health claims or indicate “free-from” certain ingredients like GMOs. These claims are often misleading when products contain such free-from labels when that product does not contain a GMO ingredient in the first place. Labels like these confuse and do not help the consumer to distinguish between scientific fact and scientific fiction. Thus, a potential consequence of marketing-based decisions with respect to labels is the potential negative influence on public perceptions around GMOs which can exacerbate the gap between public perceptions and science. The market for GMO-free labels on food has stayed on a consistent upward path. Growing demand for non-GMO products (or products labeled as such) suggests that there continues to be a belief that GMO food is unsafe or unhealthy. One objective of US NBFDS is to provide a consistent way for consumers to differentiate foods containing GMOs from those that do not.

Finally, the current study, along with previous work,^21^ raises the question of how things may potentially play out for other products or technologies, now and in the future. It will be interesting to see what other products or technologies will follow a similar pathway. In the future this pattern of events may reveal itself as new product labels are developed to meet the demand of consumers. For example, “climate-smart” labels, carbon footprint labels, pollinator-friendly claims, or other secondary standard claims which receive public attention.^73^

## Data Availability

The data that support the findings of this study are available from BuzzSumo™, Mintel™, and Overton™ but restrictions apply to the availability of these data, which were used under license for the current study, and raw data are not publicly available. Data are however available from the authors upon request and with permission of these vendors.
